# A decade of clinical negligence in ophthalmology

**DOI:** 10.1186/1471-2415-7-20

**Published:** 2007-12-20

**Authors:** Nadeem Ali

**Affiliations:** 1 Royal Victoria Infirmary, Queen Victoria Road, Newcastle-upon-Tyne, NE1 4LP, UK

## Abstract

**Background:**

To present an overview of the clinical negligence claims for ophthalmology in the National Health Service (NHS) in England from 1995 to 2006. To compare ophthalmic subspecialties with respect to claim numbers and payments.

**Methods:**

All the claims on the NHS Litigation Authority database for ophthalmology for the period 1995 to 2006 were analysed. Claims were categorised by ophthalmic subspecialty, and subspecialties were ranked according to numbers of claims, total damages paid, average level of damages and paid:closed ratio (a measure of the likelihood of a claim resulting in payment of damages).

**Results:**

There were 848 claims, 651 of which were closed. 46% of closed claims resulted in payment of damages. The total cost of damages over the period was £11 million. The mean level of damages was £37,100. Cataract made up the largest share of claims (31%), paediatric ophthalmology had the highest mean damages (£170,000), and claims related to glaucoma were most likely to result in payment of damages (64%).

**Conclusion:**

Clinical negligence claims in ophthalmology in England are infrequent, but most ophthalmologists will face at least one in their career. Ophthalmic subspecialties show marked differences with regard to their litigation profiles. From a medical protection perspective, these results suggest that indemnity premiums should be tailored according to the subspecialty areas an ophthalmologist is involved in.

## Background

Ophthalmic subspecialties differ significantly from each other in terms of disease conditions, demographics, treatments, and patient expectations. From a medicolegal perspective, this diversity means that ophthalmic subspecialties should be considered separately, rather than grouped together. Very little has been published in the field of ophthalmic negligence, however, which aims to compare and contrast between the clinically recognised ophthalmic subspecialties.

An ideal data source which may be used to study ophthalmic subspecialty litigation is the claims database of the National Health Service Litigation Authority (NHSLA). The NHSLA handles all claims against National Health Service (NHS) trusts in England [1]. Its database includes claims from April 1995 onwards. From 1995 to 2002, some trusts handled smaller claims by themselves, but the NHSLA estimates that over 90% of claims in that period reached the database (personal communication). Since April 2002, the NHSLA handled every clinical negligence claim in the NHS, and so the database from that date is all-inclusive.

Although there have been reports about ophthalmic litigation in USA, data from a European perspective are generally lacking [2]. Previous UK studies which analysed claims in the private sector represent a different spectrum of practice from the mainstream NHS [3,4]. Studies which have utilised NHSLA data for ophthalmology have been confined to a single subspecialty [5,6]. Furthermore, there are very few reports at all which seek to compare between ophthalmic subspecialties. Those that did, have utilised classifications that do not correspond to the ophthalmic subspecialties found in clinical practice [7]. Drawing on the NHSLA database, this study aims to provide a decade-long overview of ophthalmology negligence claims in the NHS in England, focusing on comparisons between the standard ophthalmic subspecialties.

## Methods

Data on ophthalmology-related claims in the NHS were obtained by submitting an online data request form to the NHSLA. Information was requested on all claims arising from the specialty of ophthalmology from 1995 to 2006. The anonymised, tabulated summary data provided by the NHSLA were then analysed and claims were grouped according to the following ophthalmic subspecialty divisions: cataract, cornea (including external eye disease), oculoplastics (including lacrimal and orbital), uveitis, glaucoma, vitreo-retinal (VR) surgery, medical retina, neuro-ophthalmology, strabismus, paediatric ophthalmology (paediatrics), oncology, and trauma. There was also a miscellaneous category for claims where a subspecialty could not be determined, as well as claims unrelated to a subspecialty (eg falls in the department).

For the whole ophthalmology data set, and for each ophthalmic subspecialty, the number of ongoing claims (open), completed claims (closed), and claims with payment of damages (paid) were determined. The total and mean level of damages, as well as the paid:closed ratio (which indicates the likelihood of a claim resulting in payment of damages) were calculated for each ophthalmic subspecialty. Subspecialties were ranked according to number of claims, total damages, mean level of damages and paid:closed ratio.

In line with NHSLA guidance, the completed study was submitted to a Risk Manager at the NHSLA for approval and comments.

## Results

The total number of claims from ophthalmology in the NHS in England for the period April 1995 to August 2006 was 848. This implies a mean annual incidence of around 75 claims per year. Over the same period, the total number of claims handled by the NHSLA for all specialties was 34,497, of which 13,449 were from surgical specialties [8]. Ophthalmology claims thus accounted for around 2.5% of the total number of claims, and 6.3% of the surgery claims over this period.

Of the 848 claims from ophthalmology, 651 were closed and 197 remained open. Of the 651 closed claims, 299 resulted in payments, giving a paid:closed ratio of 46%. The total damages for the 299 paid claims were £11.1 million over the whole period, or £982,000 per year. The mean damages for a paid claim was £37,100.

### Numbers of claims

In Table 1, the ophthalmic subspecialties are ranked by numbers of claims. Cataract accounts for nearly a third of claim numbers, with retinal specialties (VR and medical retina) comprising the next largest grouping.

**Table 1:** Ophthalmic subspecialties ranked by numbers of claims

Subspecialty	Number of claims	% of total
Cataract	264	31%
VR	81	10%
Medical retina	64	8%
Cornea	53	6%
Glaucoma	43	5%
Oculoplastics	42	5%
Neuro-ophthalmology	40	5%
Paediatrics	33	4%
Trauma	32	4%
Uveitis	28	3%
Strabismus	23	3%
Oncology	9	1%
		
Miscellaneous	136	16%

### Total damages

In Table 2, the ophthalmic subspecialties are ranked by total damages. Five subspecialties (cataract, glaucoma, medical retina, paediatrics and neuro-ophthalmology) together account for 69% of the total damages for the specialty. The lowest ranking five specialties, however, (strabismus, oncology, trauma, cornea and oculoplastics) are responsible for only 8% of the total damages.

**Table 2:** Ophthalmic subspecialties ranked by total damages

Subspecialty	Total damages (£)	% of total
Cataract	1.9 million	17%
Glaucoma	1.8 million	16%
Medical retina	1.6 million	14%
Paediatrics	1.4 million	12%
Neuro-ophthalmology	1.1 million	10%
VR	750,000	7%
Uveitis	540,000	5%
Oculoplastics	350,000	3%
Cornea	270,000	2%
Trauma	190,000	2%
Oncology	140,000	1%
Strabismus	20,000	0.1%
		
Miscellaneous	1.1 million	10%

### Mean level of damages paid

In Table 3, the ophthalmic subspecialties are ranked by mean level of damages (in paid cases). Paediatric ophthalmology has a level of mean damages over twice that of any other subspecialty. Cataract claims, though frequent, do not result in high payments.

**Table 3:** Ophthalmic subspecialties ranked by mean level of damages

Subspecialty	Mean damages (£)
Paediatrics	170,000
Glaucoma	77,000
Uveitis	68,000
Neuro-ophthalmology	63,000
Medical retina	57,000
Oncology	47,000
VR	27,000
Cataract	20,000
Oculoplastics	20,000
Trauma	19,000
Cornea	13,000
Strabismus	10,000
	
Miscellaneous	29,000

### Paid:closed ratio

In Table 4, the ophthalmic subspecialties are ranked by the paid:closed ratio, a measure of the likelihood of a claim resulting in payment of damages. There is a more even distribution among subspecialties for this parameter than for claim numbers, total damages, or mean level of damages.

**Table 4:** Ophthalmic subspecialties ranked by the paid:closed ratio

Subspecialty	Paid:closed ratio
Glaucoma	64%
Neuro-ophthalmology	58%
Oculoplastics	58%
Medical retina	52%
Cornea	50%
Paediatrics	47%
Cataract	46%
Trauma	45%
VR	40%
Uveitis	33%
Oncology	33%
Strabismus	25%
	
Miscellaneous	37%

## Discussion

This study provides an overview of clinical negligence claims in ophthalmology in England from 1995 to 2006 within the NHS. The main findings of this study can be summarised as follows. In the context of NHS litigation as a whole, ophthalmology accounts for only 2.5% of the total number of clinical negligence claims. The total cost to the NHS of damages for ophthalmology claims was £11 million in the last decade. Cataract accounts for nearly a third of ophthalmology claims, with retinal subspecialties making up nearly 20%. In terms of total damages paid, however, cataract is only slightly greater than glaucoma, medical retina, paediatrics and neuro-ophthalmology. Paediatrics has the highest level of damages, over twice that for every other subspecialty. Cataract damages, although frequent, are less costly than the average for the specialty. The likelihood of a claim resulting is payment of damages is highest for glaucoma, followed by neuro-ophthalmology and oculoplastics.

The principal strength of the study is that the NHSLA data are prospective and virtually comprehensive. This is because all claims in the NHS in England are, as a matter of procedure, handled by the NHSLA. The main limitation of the data is the brevity of the case descriptions supplied, which limits detailed analysis of causative factors for each claim. This also accounts for a large proportion of the claims which were classified in the miscellaneous category. Further limitations are that the data do not include claims from outside the NHS (ie private practice and general practice), and are confined to England.

From a risk management perspective, it is important to highlight that the negligence claims discussed in this study represent just the tip of the risk iceberg. Near-misses, undetected adverse outcomes, cases in which patients do not take matters further, and resolution of complaints by local or national non-legal bodies represent a large pool of clinical incidents, most of which never reach the NHSLA.

Most of the reports about ophthalmic litigation in the literature come from USA. These include the publications of large medical insurance groups such as the Ophthalmic Mutual Insurance Company (OMIC) and the Physicians Insurance Association of America (PIAA). The PIAA is an association of over 50 medical malpractice insurance companies, which between them insure over 60% of private practitioners in USA. The PIAA's summary data of claims for ophthalmology from 1985–2005 [9] provide a useful point of comparison with the NHSLA data. The relative contribution of ophthalmology to medical litigation is remarkably similar in England and USA (NHSLA: 2.5% of total claims; PIAA: 2.9%). The average level of damages awarded, though, is much higher in USA (PIAA: $174,000; NHSLA: £37,100). The proportion of claims resulting in payment (the paid:closed ratio), however, is higher in England (NHSLA: 46%; PIAA 29%).

Comparisons of claims between ophthalmic subspecialties in the literature are much less complete than the present study, and have used categorisations more of interest to insurers than to doctors [7]. Certain points of agreement, however, can be realised. Due to its high volume, cataract is consistently found to be the largest single contributor to claim numbers, accounting for 31% of the NHSLA claims. Cataract accounted for 47% of the PIAA claims and 29% of an older series from New Jersey [10]. In a personal review of 700 medicolegal cases, cataract was the most frequent (22% of total) [7]. A recent review of claims in the private sector in the UK also found cataract to be the commonest reason for claims, making up 39% of the total [4]. The finding that paediatric ophthalmology claims result in the highest levels of payment is corroborated by a study which examined only high-payout cases in USA [11]. This reported that patients aged under ten years had the largest monetary awards. The finding that glaucoma has the highest paid:closed ratio (64%) is confirmed by the PIAA data (42%). An area of ophthalmic practice conspicuously absent from the NHSLA data is laser refractive surgery, which is scarcely performed in the NHS. This is a major contributor to litigation in private practice, however [2,4].

In 2005, there were 2,327 medical staff in ophthalmology in England, of which 820 were consultants [12]. Using the annual claim incidence reported above, and with certain assumptions to permit estimation, the chance of an ophthalmologist of any grade being subjected to a claim in a ten-year period is approximately 30%. The average doctor in ophthalmology would therefore face one claim in a thirty year career. For a consultant, there is a 90% chance of having a claim in ten years arising from a patient under his care, which equates to two claims in a twenty year career as a consultant. Claims may be infrequent on a national level, but the majority of ophthalmologists in England will face at least one claim in their NHS career. Of some comfort is the fact that most claims never reach the courtroom, with 38% being abandoned by the claimant and a further 43% settled out of court [8].

## Conclusion

Clinical negligence claims in ophthalmology in England are reassuringly infrequent, but most ophthalmologists will face at least one in their career. Ophthalmic subspecialties show marked differences with regard to their litigation profiles. Cataract has the most claims, paediatric ophthalmology the highest level of damages, and glaucoma the highest rate of claim success. From a medical protection perspective, these results suggest that indemnity premiums should be tailored according to the subspecialty areas an ophthalmologist is involved in.

## Competing interests

The author(s) declare that they have no competing interests.

## Authors' contributions

NA devised and conducted the study, and wrote the paper.

## Pre-publication history

The pre-publication history for this paper can be accessed here:


